# Precision Genome Engineering for the Breeding of Tomatoes: Recent Progress and Future Perspectives

**DOI:** 10.3389/fgeed.2020.612137

**Published:** 2020-12-15

**Authors:** Tien Van Vu, Swati Das, Mil Thi Tran, Jong Chan Hong, Jae-Yean Kim

**Affiliations:** ^1^Division of Applied Life Science (BK21 Four Program), Plant Molecular Biology and Biotechnology Research Center, Gyeongsang National University, Jinju, South Korea; ^2^National Key Laboratory for Plant Cell Biotechnology, Agricultural Genetics Institute, Hanoi, Vietnam; ^3^Crop Science and Rural Development Division, College of Agriculture, Bac Lieu University, Bac Lieu, Vietnam; ^4^Division of Life Science, Gyeongsang National University, Jinju, South Korea

**Keywords:** CRISPR/Cas, gene editing, precision genome engineering, tomato breeding, precision breeding, new plant breeding techniques

## Abstract

Currently, poor biodiversity has raised challenges in the breeding and cultivation of tomatoes, which originated from the Andean region of Central America, under global climate change. Meanwhile, the wild relatives of cultivated tomatoes possess a rich source of genetic diversity but have not been extensively used for the genetic improvement of cultivated tomatoes due to the possible linkage drag of unwanted traits from their genetic backgrounds. With the advent of new plant breeding techniques (NPBTs), especially CRISPR/Cas-based genome engineering tools, the high-precision molecular breeding of tomato has become possible. Further, accelerated introgression or *de novo* domestication of novel and elite traits from/to the wild tomato relatives to/from the cultivated tomatoes, respectively, has emerged and has been enhanced with high-precision tools. In this review, we summarize recent progress in tomato precision genome editing and its applications for breeding, with a special focus on CRISPR/Cas-based approaches. Future insights and precision tomato breeding scenarios in the CRISPR/Cas era are also discussed.

## Introduction

The domestication of wild plants, in which plant parts or seeds with desirable characteristic(s) are kept for the next cropping seasons, is the first step of plant breeding (Lin et al., 2014). Post-domestication, the selection of more desirable traits from the domesticated plants can generate novel varieties with added value. Traditionally, plant breeding approaches have been based on the selection of visibly desirable traits from the cultivated crops. This practice was subsequently extended to the selection of offspring from two distinct parental plants, in the so-called cross-breeding or hybrid crossing technique, after Mendel discovered phenotype-associated alleles and genetic inheritance rules during cross-pollination of pea plants. The process is time-consuming and laborious (Bai and Lindhout, 2007). In the modern era, the selection of desirable traits that usually links to one or several quantitative trait loci (QTL) has been assisted by molecular markers, thereby shortening cross-breeding time and labor (Collard and Mackill, 2008; Foolad and Panthee, 2012). One of the major limitations to the traditional crossbreeding technique is linkage drag, which can introduce undesirable traits from a parental donor in addition to the desirable ones. Genetic engineering approaches, such as transgenesis, have efficiently helped overcome this limitation by introducing only the genes/alleles of interest into an elite plant. However, due to the need to introduce a selection marker, which is usually isolated from non-plant sources, or the *de novo* integration of single or multiple copies of foreign DNA into a targeted plant, the products of the process have been tightly regulated and require many lengthy and costly trials and biosafety assessments before the strain can be released to the environment (Bai and Lindhout, 2007).

Another major technology for crop breeding is the generation of random mutations in a plant by chemicals or physical agents, such as gamma rays. The chemicals or radiation randomly induces large amounts of DNA damage in the genome of a plant, such as nucleotide chemical modifications or double-stranded breaks (DSBs), thereby generating many mutant strains. Extensive screening and selection of the mutants are required to obtain a plant with traits of interest. However, although time-consuming and repetitive, back crossing is often needed to remove non-desirable mutations, and many unexpected modifications may also be fixed in the genome of the mutant plants (Shelake et al., 2019). Nevertheless, plants generated by random mutation approaches have been as accepted as those from conventional breeding approaches. Recently, new plant breeding technologies (NPBTs), especially clustered regularly interspaced short palindromic repeats (CRISPR)/CRISPR-associated protein (Cas)-based approaches, have been emerging as the superior precision plant breeding technologies for crop genetic improvement and bringing hope to our future agriculture (Belhaj et al., 2013; Chen et al., 2019). The power of CRISPR/Cas systems is the ability to specifically introduce theoretically any genetic modification of interest to any genomic site of a plant without any linkage drag phenomenon. We can now edit the genomes of crops by native CRISPR/Cas complexes, including the induction of targeted single base transition/transversion by a range of base editors, customization of DNA changes by prime editors, or precise replacement of a single base to several kilobases by homologous recombination (HR)-based knock-in (HKI) with the assistance of the CRISPR/Cas system (Cermak et al., 2015; Rees and Liu, 2018; Chen et al., 2019; Lin et al., 2020; Vu et al., 2020). All of these approaches are site-specific and can be controlled to be free of off-target or transgene effects. At the targeted sites, the levels of precise sequence modifications are determined by the DNA repair pathway that is directed to repair DNA damage induced by the CRISPR/Cas complex.

The above mentioned NPBTs have been successfully adapted for tomato genome editing and the subsequent precision breeding of new varieties without linkage drag. Appropriate applications of the NPBTs also helped accelerate the introgression of novel traits from wild relatives of tomato into their elite cultivars and made it feasible with *de novo* domestication of wild tomato (Zsogon et al., 2017, 2018; Li et al., 2018c). In this review, we summarize recent progress in precision breeding of tomato using CRISPR/Cas-based approaches and further discuss the future perspectives within this field.

## Current Status of Tomato Breeding

### Conventional Approaches

The domestication of the cultivated tomato (*Solanum lycopersicum* var. *lycopersicum*) is believed to have started in the Andean region of Central America and has undergone two intermediate stages, represented first by *S. pimpinellifolium* and then by *S. lycopersicum* var. *cerasiforme* as the direct ancestor. During domestication, the evolution/selection force was fruit size (Lin et al., 2014). The other characteristics that can be used to distinguish domesticated tomato and its wild relatives are growth parameters and other fruit traits (Bai and Lindhout, 2007). The modern cultivated tomato was thought to have initially spread to the Old World from Mexico to Europe and later from Europe to the rest of the world (Jenkins, 1948). Following the spreading and selection of tomato varieties for adaptation to each specific geographical area, the genetic diversity of the domesticated tomato has been substantially reduced thanks to genetic drift (Bai and Lindhout, 2007; Lin et al., 2014).

The major conventional approaches for tomato breeding include pedigree, hybrid, and backcross breedings that focus on combinations of various traits for different consumption and market requirements (Bai and Lindhout, 2007). Tomato breeders can be individuals such as farmers or institutions (public and private sectors) and may have different goals for breeding programs. Due to the reduced genetic diversity among inbred populations of tomato resulting from the long period of selective domestication (Ranc et al., 2008), cross-hybridization among the populations is the simplest and fastest way to obtain genetic variations and subsequent selection of new varieties exhibiting novel traits. The pedigree method keeps performance records of all the progenies in many generations of a hybrid from genetically distant parents, thereby supporting the selection of varieties with new traits of interest. The new traits can arise only from the gene pools of the parental populations. To obtain novel traits, such as biotic or abiotic stress tolerance, from wild relatives, tomato breeders use a backcross breeding method to introgress new alleles into cultivated lines and recurrently backcross progenies with the parental cultivated lines to recover their genetic backgrounds. These conventional breeding approaches require extensive observation and selection of the best progenies in many generations and are therefore time-consuming and laborious.

In the genomics era, the selection of specific genotypes can be assisted by molecular markers through so-called marker-assisted selection (MAS) (Collard and Mackill, 2008). Usually, DNA markers that are tightly linked to the QTLs of interest are used to track the presence of QTLs in hybrid offspring by PCR or sequencing. With the ability to sequence the whole genome of a plant at minimal cost, plant breeding by the conventional method has become much more efficient.

### New Plant Breeding Approaches

NPBTs represent the newly emerging molecular techniques applied to plant breeding in the genomics and genome editing era, including the CRISPR/Cas nucleases. The NPBTs emphasize engineering plant genomes with a high degree of precision. In particular, with the revolutionary advent and applications of CRISPR/Cas systems for plant genome engineering, plant breeding at the molecular level has become more efficient and precise (Chen et al., 2019). With CRISPR/Cas complexes, many options are available for specifically modifying gene sequences of interest from a single base by base editors and prime editors to several kilobases with HKI (Van Vu et al., 2019).

### CRISPR/Cas-Based Genome Editing

**Figure 1:**
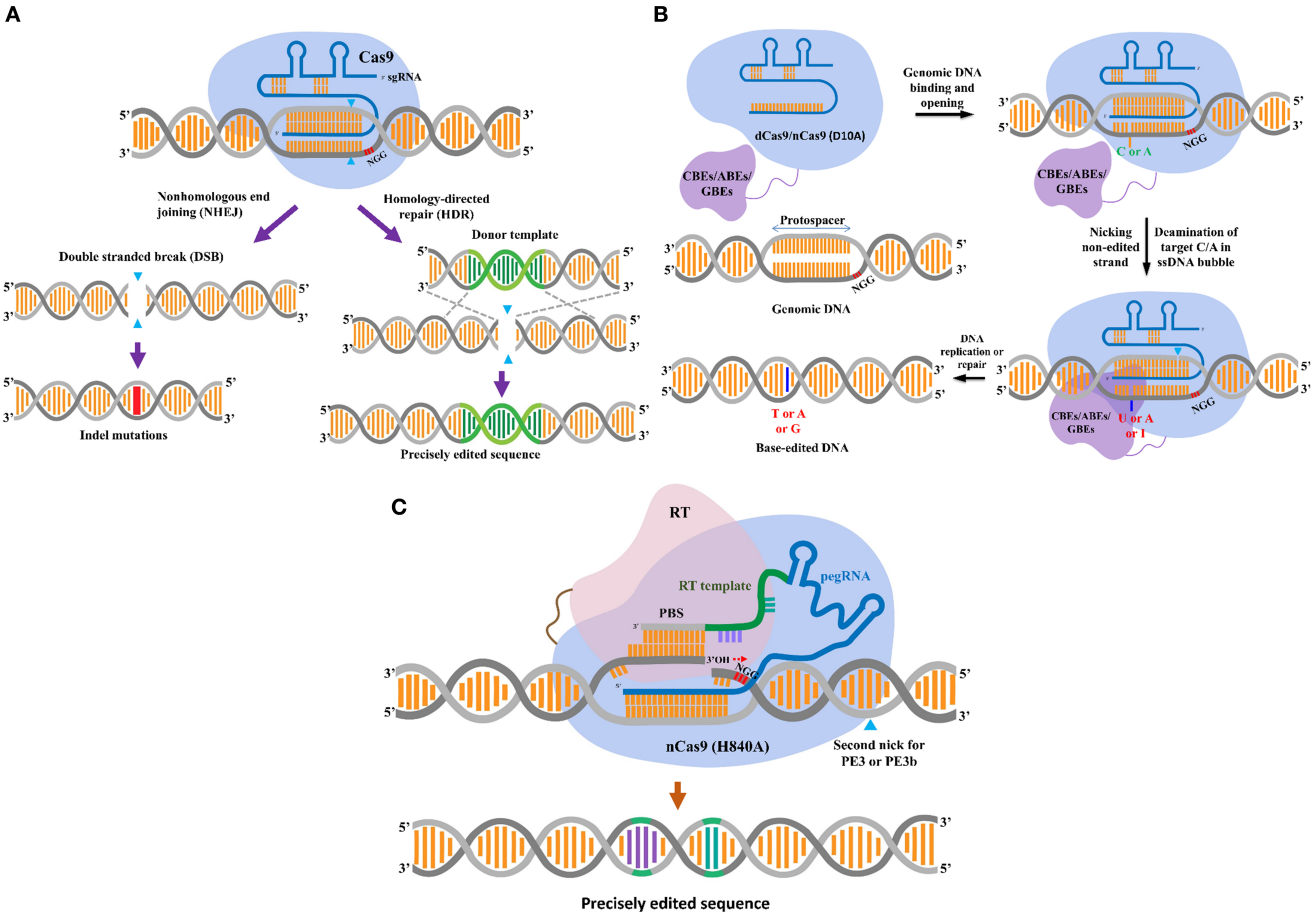
CRISPR/Cas-based plant gene-editing tools. **(A)** CRISPR/Cas9-mediated double-stranded break (DSB) repair mechanism. The CRISPR-associated enzyme Cas9-sgRNA complex breaks down the target DNA to create a DSB, that leads to gene editing via two major DSB repair pathways, i.e., non-homologous end joining (NHEJ) and homology-directed repair (HDR) pathway. The blue triangles indicate locations of the cut site. The NHEJ pathway usually results in small insertions and deletions, as indicated by the red line. The HDR pathway uses a DNA donor template with two homologous ends to the DSB terminals to precisely introduce the edited sequence (green helix) into the genomic site. **(B)** DNA base editors. From left to right and top to down: base editors are created by fusing a dead/nickase Cas9 (d/nCas9) (light blue) to a nucleobase deaminase (in case of cytosine base editors (CBEs) and adenine base editors (ABEs), painted in purple), and a uracil-DNA glycosylase (Ung) [for glycosylase base editors (GBEs)]. The d/nCas9 activated by complexing with a guide RNA (gRNA, in blue color) “scans” for and binds to an NGG PAM and its upstream adjacent sequence that is complementary to the spacer of gRNA. The d/nCas9-gRNA-DNA bound site forms an R-loop that exposes the non-complementary strand as a disordered, unprotected ssDNA. Subsequently, the fused ssDNA-specific nucleobase deaminase works on the exposed strand and deaminates its favorable substrates (C for CBEs, A for ABEs, and C/A for GBEs). In the case of nCas9, a nick would be introduced to the non-edited strand (the blue triangle) for enhancing the editing efficiency. Ultimately, CBEs convert C to T via a U intermediate; and ABEs deaminate A to inosine (I) that is recognized as a G and then fixed as a G after DNA repair or replication (highlighted as the dark blue stick). GBEs consist of a Cas9 nickase, a cytidine/adenine deaminase, and a uracil-DNA glycosylase (Ung). Ung excises the deaminated product (U/I) formed by the deaminase, creating an apurinic/apyrimidinic (AP) site that initiates the process of repairing DNA. In E. coli, the activation-induced cytidine deaminase (AID) was used to construct AID-nCas9-Ung for C to A conversion. In mammalian cells, rat APOBEC1 substituted AID (APOBEC-nCas9-Ung) for C-to-G conversions. **(C)** The prime editor. The prime editing system consists of a CRISPR/ Cas complex by fusion of reverse transcriptase (RT, pink) domain to a C-terminal of Cas 9 (H840A) nickase, and a prime editing gRNA [pegRNA, composed of a gRNA with a scaffold (blue), an RT template (green) and a prime binding sequence (PBS, gray)] with a 3′ extended PBS that binds to the 3′ nicked site of the target DNA having the PAM site. The nicked terminal's free 3′-OH provides a prime for the RT to copy the genetic information from the RT template (the orientation is indicated by the discontinuous red arrow). The copied information is then fixed into the genomic site via a complex process that includes flapping of the competitively original strand and subsequent integration of the edited strand via DNA repair that may not be fully understood in plants.

CRISPR/Cas-based DNA interference is a phenomenon of prokaryote defense against infectious phages (Barrangou et al., 2007). In general, a single-unit Cas nuclease, such as SpCas9, is activated by complexing with a single CRISPR guide RNA (sgRNA), and the Cas-sgRNA complex “scans” for a dsDNA target that contains a complementary protospacer sequence. An NGG protospacer adjacent motif (PAM, N=A, T, G or C), binds to it and then cleaves both the strands (Figure 1A) (Jinek et al., 2012). Due to the DSB-forming nature of the CRISPR/Cas nucleases, they have been used for specifically inducing targeted mutations within a genome of interest (Belhaj et al., 2013; Li et al., 2013; Nekrasov et al., 2013; Shan et al., 2013). The DNA damage triggers the repair system to maintain the integrity of the genomes, thereby avoiding the fatal effects that a single unrepaired DSB can induce. The major pathways involved in DSB repair are the non-homologous end-joining (NHEJ) and cell cycle-dependent HR pathways (Figure 1A) (Puchta, 2005; Lieber, 2010; Chapman et al., 2012). The NHEJ mechanism facilitates the repair of the two DSB terminal ends by direct ligation with the activity of DNA ligase IV. Under unfavorable conditions, NHEJ may be erroneous and hence result in small DNA mutations (i.e., deletions or insertions). In plant somatic cells, the HR pathway repairs the DSBs by recombining the sequences flanking the broken ends with homologous sequences from DNA donors. HR can be divided into at least two major subpathways: single-strand annealing (SSA) and synthesis-dependent strand annealing (SDSA) (Puchta, 2005; Van Vu et al., 2019).

Recent studies have revealed multiple CRISPR/Cas systems that can be used to edit RNA (Cox et al., 2017) or stimulate nucleobase damage by deaminases to induce single base transitions (Komor et al., 2016; Nishida et al., 2016; Gaudelli et al., 2017) or transversions (Kurt et al., 2020; Zhao et al., 2020) (Figure 1B). Another interesting approach for genome editing without inducing DSBs is a prime editor that uses a reverse transcriptase to copy genetic information from an extended sgRNA (Figure 1C). The priming extended gRNA (pegRNA) primes the Cas9-fused RT by binding to the sequence upstream of the nicked site on the untargeted sequence, thereby triggering reverse transcription from the 3′ OH of the nicked end using the pegRNA as a template (Anzalone et al., 2019). The prime editor system was successfully applied in rice and wheat (Lin et al., 2020), but it seems that the performance of the present prime editor in plants was limited. Thus, there is a research opportunity for improvement for further applications in plants (Butt et al., 2020; Hua et al., 2020).

### Tomato Precision Genome Engineering

#### CRISPR/Cas-Based Targeted Mutagenesis

**Figure 2:**
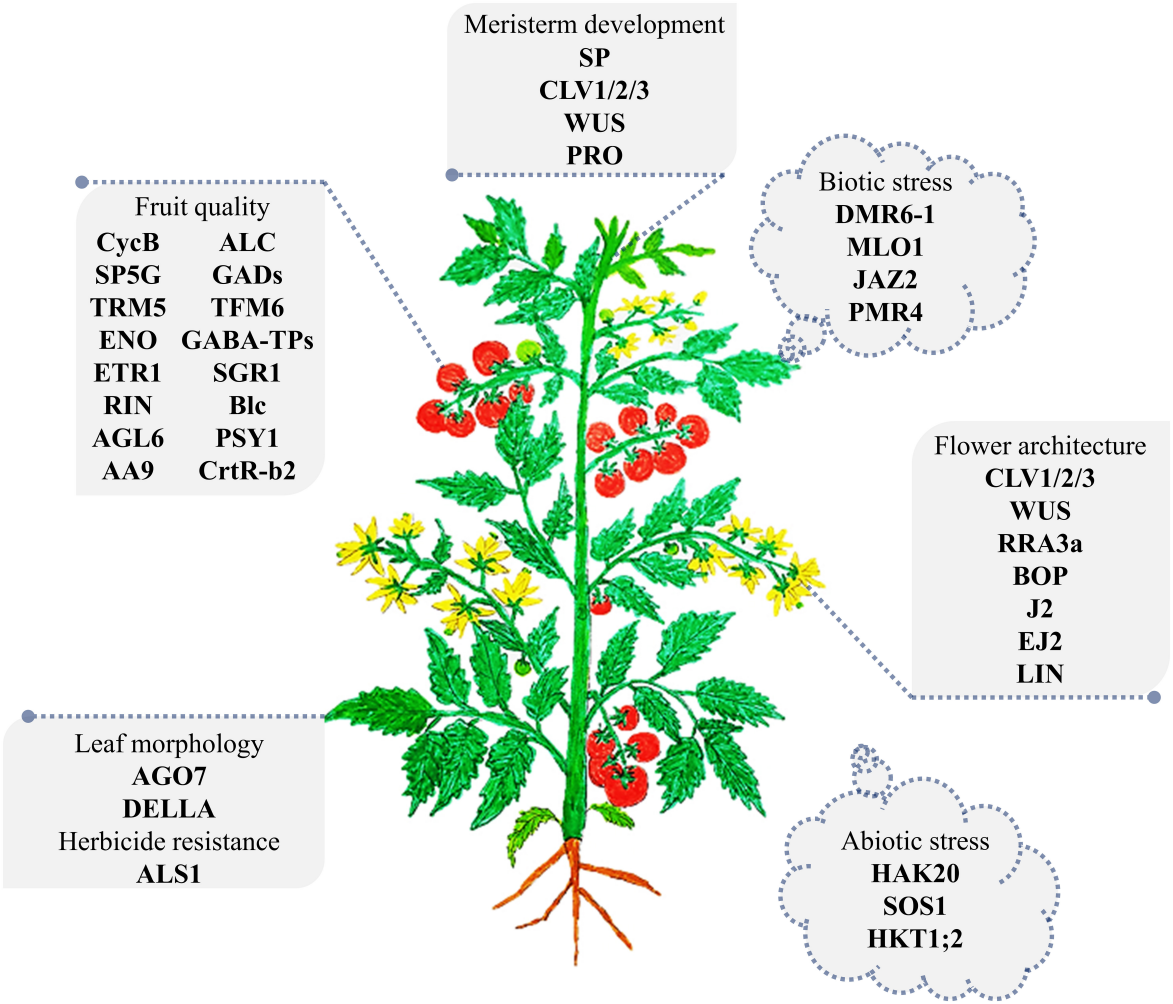
The major genes targeted by CRISPR/Cas-based targeted mutagenesis in tomato. A representative tomato plant at the vegetative growth stage was drawn. The CRISPR/Cas edited genes are illustrated in association with the organs they affect. The abiotic stress gene set is shown surrounding the plant, indicating their global impacts on the plant. The detail of related studies is summarized in Table 1. The plant is not drawn to scale.

The CRISPR/Cas revolution has paved the way for powerful precision plant breeding using molecular tools. Starting in 2013, the early publications regarding CRISPR/Cas-based targeted mutagenesis in tomato focused on the feasibility of efficient use of the tool and its applicability for studying gene function with knock-out approaches (Figure 2).

##### Engineering for Growth Habits

The first CRISPR/Cas9-based tomato genome editing data were published in 2014 by Brooks and coworkers and showed highly efficient CRISPR/Cas9-based targeted mutations of four different loci (Figure 2; Table 1). Wiry leaf phenotypes (recessive) were revealed in the first generation of transformants of which both the alleles of the tomato ARGONAUTE7 (SlAGO7) gene were mutated (Brooks et al., 2014). The genotype and phenotype were also shown to be inherited in the next generations, confirming the huge potential of CRISPR/Cas technology in tomato genetic improvement and breeding.

**Table 1:** Precision genome editing in tomato using CRISPR/Cas complexes.

**Trait category**	**Target gene**	**Accession ID**	**Editing tool**	**Major mutant phenotype**	**Repair pathway**	**Editing efficiency (%)**	**References**
**CRISPR/Cas-BASED TARGETED MUTAGENESIS**							
Growth habit	SlAGO7 (Argonaute 7)	Solyc01g010970	CRISPR/SpCas9	Typical compound flat leaves become needle like or wiry	cNHEJ	48.0	Brooks et al., 2014
	SlHPAT homolog	Solyc08g041770		Multiple aspects of tomato reproductive development		75.0	
	SlHPAT homolog	Solyc07g021170				100.0	
	SlHPAT homolog	Solyc12g044760				100.0	
	SlCLV3 (Clavata 3)	Solyc11g071380	CRISPR/SpCas9	Branched inflorescences with fasciated flowers	cNHEJ	57.1	Xu et al., 2015
	SlCLV1	Solyc04g081590		Weak branching and fasciated flowers		100.0	
	SlCLV2	Solyc04g056640		Weak branching and fasciated flowers		83.3	
	SlRRA3a (Reduced residual arabinose 3a)	Solyc04g080080		Branched inflorescences with fasciated flowers		66.7	
	SlPDS (phytoene desaturase)	Solyc03g123760	CRISPR/SpCas9	Albino	cNHEJ	71.4–100.0	Pan et al., 2016
	SlPIF4 (Phytochrome interacting factor 4)	Solyc07g043580		No obvious abnormal phenotype		84.0–89.5	
	SlBOPs (Blade-on-petiole)	SlBOP1 (Solyc04g040220), SlBOP2 (Solyc10g079460), SlBOP3 (Solyc10g079750)	CRISPR/SpCas9	Flowering defect	cNHEJ	-	Xu et al., 2016
	SlJ2 (Jointless-2)	Solyc12g038510	CRISPR/SpCas9	Jointless unbranched inflorescences	cNHEJ	-	Roldan et al., 2017; Soyk et al., 2017
	SlEJ2 (Enhancer-of-jointless2)	Solyc03g114840		Exceptionally large sepals and pear-shaped fruits		-	
	SlLIN (Long inflorescence)	Solyc04g005320		Moderately branched inflorescences and increased flower production		-	
	SlTRM5 (TONNEAU1 Recruiting Motif5)	Solyc07g008670	CRISPR/SpCas9	Slightly flatter fruit	cNHEJ	-	Wu et al., 2018
	SlENO (Excessive number of floral organs)	Solyc03g117230	CRISPR/SpCas9	Increased number of floral organs and multilocular fruits	cNHEJ	100.0	Yuste-Lisbona et al., 2020
	SlPRO (Procera)	Solyc11g011260	CRISPR/SpCas9	Dwarf/gibberellin-responsive dominant dwarf DELLA allele	cNHEJ	17.4	Tomlinson et al., 2019
Fruit quality	SlRIN (Ripening inhibitor)	Solyc05g012020	CRISPR/SpCas9	Incomplete-ripening fruits, extended shelf life	cNHEJ	11.8–50.0	Ito et al., 2015
	SlAGL6 (Agamous-like 6)	Solyc01g093960	CRISPR/SpCas9	Seedless	cNHEJ	-	Klap et al., 2017
	SlIAA9 (Auxin-induced 9)	Solyc04g076850	CRISPR/SpCas9	Seedless	cNHEJ	42.9–100.0	Ueta et al., 2017
	SlALC (alcobaca)	FJ404469	CRISPR/SpCas9	Long-shelf life	cNHEJ	72.7	Yu et al., 2017
	SlGAD2 (glutamate decarboxylase 2)	B1Q3F1	CRISPR/SpCas9	Increased GABA accumulation: In T0 fruits: 1.5–10.0 folds	cNHEJ	68.8–78.6	Nonaka et al., 2017
	SlGAD3 (glutamate decarboxylase 3)	B1Q3F2		Increased GABA accumulation: In T0 fruits: 1.0–5.0 folds In T1 fruits: 7.0–15.0 folds		25.7	
	SlTFM6 (Tomato fruit malate on chromosome6)	Solyc06g072910	CRISPR/SpCas9	Reduced fruit malate content.	cNHEJ	-	Ye et al., 2017
	SlGABA-TP1 (pyruvate-dependent γ-aminobutyric acid transaminase 1)	AY240229	CRISPR/SpCas9	2.85-fold increased GABA accumulation	cNHEJ	50.0–56.8	Li et al., 2018a
	SlGABA-TP2	AY240230		-		0.0	
	SlGABA-TP3	AY240231		3.5-fold increased GABA accumulation (together with SlGABA-TP1 mutation)		46.6	
	SlCAT9 (Cationic amino acid transporter 9)	XM_004248503		No fruit		6.8	
	SlSSADH (Succinate semialdehyde dehydrogenase)	NM_001246912		No fruit		9.1	
	SlSGR1 (Stay green 1)	DQ100158	CRISPR/SpCas9	5.1-fold increase of lycopene content in fruit	cNHEJ	41.7–95.8	Li et al., 2018c
	SlLCY-E (Lycopene ε-cyclase)	EU533951		-		8.3	
	SlBlc (Beta-lycopene cyclase)	XM_010313794		1.83-fold increase of lycopene content in fruit		91.7	
	SlLCY-B1 (Lycopene β-cyclase 1)	EF650013		-		0.0	
	SlLCY-B2 (Lycopene β-cyclase 2)	AF254793		-		4.2	
	SlPSY1 (Phytoene synthase 1)	P08196	CRISPR/SpCas9	Yellow-flesh fruit	cNHEJ	75.0–84.0	D'Ambrosio et al., 2018
	SlCrtR-b2 (Beta-carotene hydroxylase 2)	Q9S6Y0		White-flower		75.0–100.0	
Biotic stress tolerance	SlDMR6-1 (Downy mildew resistance 6-1)	Solyc03g080190	CRISPR/SpCas9	Disease resistance against different pathogens, including *P. syringae, P. capsici*, and *Xanthomonas* spp.	cNHEJ	-	Paula de Toledo Thomazella et al., 2016
	SlMLO1 (Mildew Locus O 1)	Solyc04g049090	CRISPR/SpCas9	Powdery mildew disease resistance	cNHEJ	80.0	Nekrasov et al., 2017
	SlJAZ2 (Jasmonate Zim Domain 2)	Solyc12g009220	CRISPR/SpCas9	Resistance against *P. syringae* pv. tomato DC3000	cNHEJ	65.2	Ortigosa et al., 2019
	SlPMR4 (Powdery Mildew Resistance 4)	Solyc07g053980	CRISPR/SpCas9	Reduced susceptibility to Powdery mildew disease	cNHEJ	45.9	Santillan Martinez et al., 2020
Abiotic stress tolerance	SlMAPK3 (Mitogen activated protein kinase 3)	AY261514	CRISPR/SpCas9	Reduced drought tolerance	cNHEJ	41.8	Wang et al., 2017
	SlNPR1 (Nonexpressor of pathogenesis-related gene 1)	KX198701	CRISPR/SpCas9	Reduced drought tolerance	cNHEJ	33.3–46.7	Li et al., 2019
	SlHAK20 (High-affinity K+ 20)	Solyc04g008450	CRISPR/SpCas9	Hypersensitivity to salt stress	cNHEJ	-	Wang et al., 2020a
	SlSOS1 (Salt Overly Sensitive 1)	Solyc11g044540	CRISPR/SpCas9	Increased salt sensitivity	cNHEJ	-	Wang et al., 2020b
	SlCBF1 (C-repeat/dehydration-responsive element binding factor 1)	AAS77820	CRISPR/SpCas9	Reduced chilling tolerance	cNHEJ	25.0–57.5	Li et al., 2018b
	SlBZR1 (brassinazole-resistant 1)	Solyc04g079980	CRISPR/SpCas9	Reduced heat tolerance	cNHEJ	-	Yin et al., 2018
	SlHyPRP1 (Hybrid proline-rich protein 1)	Solyc12g009650	CRISPR/SpCas9	Salinity tolerance	cNHEJ	4.5–20.0	Tran et al., 2020
**CRISPR/Cas-BASED PRECISE DNA CHANGES/REPLACEMENTS**							
Growth habit	SlDELLA	Solyc11g011260	nSpCas9-PmCDA1	Reduced serrated leaflets	BER/NER	54.5	Shimatani et al., 2017
	SlETR1 (Ethylene receptor 1)	Solyc12g011330	nSpCas9-PmCDA1	Insensitivity to ethylene	BER/NER	70.0	
Growth habit	SlANT1 (Anthocyanin 1)	Solyc10g086260	CRISPR/SpCas9	Global anthocyanin over-accumulation	HDR	11.7	Cermak et al., 2015
Growth habit	SlANT1	Solyc10g086260	CRISPR/LbCas12a	Global anthocyanin over-accumulation	HDR	12.8	Vu et al., 2020
Abiotic stress tolerance	SlHKT1;2 (High-affinity potassium transporter 1;2)	Solyc07g014680		Salinity tolerance		0.7	
Herbicide resistance	SlALS1 (Acetolactate synthase 1)	Solyc03g044330	nSpCas9-PmCDA1	Chlorsulfuron resistance	BER/NER	71.0	Veillet et al., 2019
Herbicide resistance	SlALS1	Solyc03g044330	CRISPR/SpCas9	Chlorsulfuron resistance	HDR	12.7	Danilo et al., 2019

The regulation of plant growth and inflorescence development for higher yield and better fruit production has been a traditional priority in tomato domestication and breeding. In 2015, CRISPR/Cas9 was used in a functional study of novel genes involved in the Clavata (CLV)-Wushel (WUS) circuit in the regulation of shoot meristem size. Targeted mutagenesis of tomato CLV homologs and an arabinosyltransferase generated mutant plants with phenotypes resembling that of natural mutants (Table 1). The work advanced our knowledge about the regulation of the CLV pathway, which may be very helpful in the customization of mutant alleles for tomato breeding (Xu et al., 2015). Efficiently targeted knock-out of SlPDS produced biallelic KO allele-carrying strains with an albino phenotype as a visible marker but also led to the suppression of their growth (Pan et al., 2016). Genes involved in inflorescence growth and maturation were also extensively studied using CRISPR/Cas9-based targeted mutagenesis (Xu et al., 2016; Roldan et al., 2017; Soyk et al., 2017). Inflorescence maturation was positively linked to the tomato BLADE-ON-PETIOLE transcriptional cofactors (SlBOPs). SlBOP knock-out mutants showed flowering defects as they produced inflorescences with only a single flower (Xu et al., 2016). Further investigations into the inflorescence structure, growth, and maturation led by Soyk et al. shed light on the roles of the other MADS-box transcription factors in these processes. The J2, EJ2, and LIN genes play roles in controlling inflorescence branching and hence flower numbers in a quantitative manner (Roldan et al., 2017; Soyk et al., 2017). Dosing the mutations of the genes by combining their mutated alleles in various groupings may help to design desirable inflorescences for production goals (Soyk et al., 2017). During fruit setting, the fruit shape is determined by the activity of several proteins, including OVATE and SUPPRESSOR OF OVATE1 (SOV1), members of the OVATE FAMILY PROTEIN (OFP) family. Mutated alleles of OVATE and SOV1 led to the production of elongated fruits (oval or pear shape), and the shape could be rescued by knocking out TRM5, a member of the TONNEAU1 Recruiting Motif (TRM) family, which was shown to work downstream and in close contact with *ovate* and *sov1* (Wu et al., 2018). The floral organ and locule number were also enhanced in the KO mutant of EXCESSIVE NUMBER OF FLORAL ORGANS (SlENO). CRISPR/Cas9-based targeted mutagenesis of the SlENO locus was extremely efficient (Yuste-Lisbona et al., 2020). Plant vegetative growth could be regulated by gibberrellins (GAs) via their interaction with DELLA protein, a negative regulator of plant growth, thereby subjecting it to degradation. A mutation in the interacting site of PROCERA, a DELLA protein, blocked GA binding and thus suppressed plant growth, resulting in tomato dwarfism (Tomlinson et al., 2019).

##### Fruit Quality Improvement

Tomato fruit quality is increasing of interest in modern markets, especially for fresh uses. However, cultivated tomatoes have lost many fruit qualitative characteristics, such as flavors (Tieman et al., 2017), due to the yield-based domestication and industrialization of tomato production. Studying the gene functions and their mutants in the fruit setting and maturation is important for customizing tomato fruit for higher quality by CRISPR/Cas9 technology (Figure 2). Fruit shelf life is an important parameter of fruit quality and storage capability. A MADS-box transcription factor gene (SlRIN, Table 1) involved in fruit ripening was destroyed by CRISPR/Cas9, resulting in an incomplete ripening stage and extended shelf life (Ito et al., 2015). However, the *rin* mutants, even in the heterozygous form, may lead to a reduction of lycopene content in tomato fruits (Herner and Sink, 1973). Another well-characterized mutant allele in tomato breeding for long shelf life is *alc* (*alcobaca*), which may introduce fewer side effects (Yu et al., 2017). The seedless tomato is an interesting example with fresh uses as well as uses in processing. The parthenocarpic tomato lines were created by EMS mutagenesis of the tomato AGAMOUS-LIKE 6 (SlAGL6) gene that promoted fruit development without fertilization, especially under heat stress. The *slagl6* allele could also be specifically generated with CRISPR/Cas9 complexes. Interestingly, if fertilization is successful under normal conditions, then seeds are still normally developed, suggesting that the traits could be used in practical breeding (Klap et al., 2017). An alternative approach for breeding of parthenocarpic tomato was through targeted mutation of AUXIN-INDUCED 9 (SlIAA9), a repressor of fruit development without fertilization. Knock-out mutation of SlIAA9 led to the production of parthenocarpic fruits but also abnormal leaf morphology (Ueta et al., 2017) that could ultimately affect yield. Malic acid (malate salt) is an intermediate metabolite of C4 plants, and it plays roles in plant growth, fruit quality, and Al detoxification in roots (Ye et al., 2017). Tomato fruit malate on chromosome6 (TFM6) was shown to be associated with tomato fruit malate content. CRISPR/Cas9-based deletion of the TFM6 gene led to a reduction in fruit malate content. However, a 3-bp deletion in the binding site of a WRKY transcription repressor (SlWRKY42) in the promoter of TFM6 enhanced the accumulation of malate in fruit (Ye et al., 2017).

ɤ-Aminobutyric acid (GABA), a non-protein amino acid, acts as an inhibitory neurotransmitter and hypotensive relief factor that benefits human health (Takayama et al., 2015; Li et al., 2018a). However, even produced at relatively high levels compared to other crops, the GABA content in tomato fruits is still very low. To enhance GABA production in tomato fruits, Nonaka and coworkers used CRISPR/Cas9 to target the autoinhibitory C-terminal coding sequences of two genes, glutamate decarboxylase 2 and 3 (SlGAD2 and SlGAD3, respectively), that are involved in GABA synthesis in fruit stages. The SlGAD3 mutated plants carrying a premature stop codon before the autoinhibitory domain produced GABA in T1 fruits at 7–15-fold higher levels than non-edited plants (Table 1) (Nonaka et al., 2017). Lee and coworkers generated hybrid lines of the best event (TG3C37) obtained from the work of Nonaka et al. The heterozygous state of the alleles also accumulated a high level of GABA in fruits while having minimal effects on plant growth and fruit development (Lee et al., 2018). A similar approach was also taken by Li and coworkers, but they targeted five genes (Table 1) involved in the GABA conversions of the GABA shunt. The highest accumulation of GABA in red fruits was 3.5-fold higher than that of WT fruits (Li et al., 2018a). However, higher GABA accumulation in leaves (~6–20 folds) (Li et al., 2018a), and fruits (11–12 folds, transgenic plants over-expressing C-terminal truncated SlGAD3 in fruits) (Takayama et al., 2017) led to reduced growth and prolonged flowering time, and changing fruit contents, respectively. Two possible explanations, which are not mutually exclusive, are currently available. The first hypothesis is that GABA overaccumulation causes a deficit of glutamate, the precursor of GABA. The second explanation is that GABA itself functions in the plant signaling pathway; thus, its overaccumulation is detrimental to plant growth. Therefore, together with GABA overaccumulation, strategies to enhance the glutamate synthesis pathway or sequester GABA into vacuoles might be future options.

Carotenoids produced during tomato fruit growth and maturation contribute to a large portion of fruit pigments and volatiles as the byproducts of their metabolism (metabolites). Lycopene, produced in tomato fruit, is a carotenoid that contributes to the color of the fruit and is hypothesized to possess potential health effects (Story et al., 2010). Therefore, tomato breeding for enhancement of lycopene accumulation in fruit is of interest. In total, five genes involved in lycopene metabolism at the early processes (SlSGR1) and lycopene cyclization stages (LCY-E, Blc, LCY-B1, and LCY-B2) were targeted for the enhancement of its production (Table 1). Up to a 5.1-fold increase in lycopene content was recorded after the single mutation of SlSGR1, while additional mutated genes caused a reduction of the lycopene amount compared to that in the SlSGR1 mutant, though the content was still much higher compared to that of non-edited fruits (Li et al., 2018d). Regulation of carotenoid accumulation in tomato fruit has also been used to customize fruit color. Targeting the genes encoding enzymes involved in the carotenoid pathway is the major approach using CRISPR/Cas9 complexes. Targeted knock-out of the phytoene synthase 1 (SlPSY1) led to the abolishment of lycopene production and thus resulted in tomato fruits with yellow flesh (D'Ambrosio et al., 2018).

##### Biotic Stress Tolerance Enhancement

Environmental conditions are continuously changing; under the pressure of arable land shortages, sustaining food production to feed increasing populations will be a challenge by 2050 (Hickey et al., 2019). The breeding of resilient crops for stress tolerance is a major solution to help meet this challenge. CRISPR/Cas9 has been efficiently used to target genes encoding negative regulators of biotic as well as abiotic stress response pathways (Paula de Toledo Thomazella et al., 2016; Nekrasov et al., 2017; Ortigosa et al., 2019; Santillan Martinez et al., 2020) (Figure 2 and Table 1).

Bacterial speck disease caused by *Pseudomonas syringae* pv. tomato is one of the major threats to tomato production since it can lead to losses in yield and fruit quality (Cai et al., 2011). In an early application of CRISPR/Cas9 targeting a knock-out of a positive regulator of the disease, mutant alleles of a tomato ortholog of *Arabidopsis* downy mildew resistance 6 (DMR6) were generated. Initial tests of the mutants showed resistance against *P. syringae* pv. tomato DC3000 (*Pto* DC3000), *Phytophthora capsici*, and *Xanthomonas* spp. (Paula de Toledo Thomazella et al., 2016) that may be highly useful sources for tomato breeding. Another interesting approach to prevent *P. syringae* colonization was via regulation of stomata opening/closing. *P. syringae* produces coronatine (COR), a mimic of jasmonic acid-isoleucine (JA-Ile), during infection, and COR subsequently stimulates stomata opening and triggers degradation of a major COR coreceptor, JASMONATE ZIM DOMAIN 2 (JAZ2). CRISPR/Cas9-based truncation of the C-terminal Jas domain of SlJAZ2 generated enhanced *Pto* DC3000-resistant plants without altering the resistance against the pathogenic fungus *Botrytis cinerea* (Ortigosa et al., 2019). The fungal pathogen *Oidium neolycopersici* is the causal agent of powdery mildew disease, which leads to serious yield losses in tomato production and fruit quality reduction (Jones et al., 2001). Some members of the transmembrane protein Mildew Locus O (MLO) family are responsible for susceptibility to *O. neolycopersici* infection. Among the 16 MLOs in tomato, the SlMLO1 was shown to be the major susceptibility gene, and its natural loss-of-function mutants exhibited powdery mildew disease resistance (Zheng et al., 2016). CRISPR/Cas9-based mutant strains carrying homozygous *Slmlo1* alleles that are 48-bp truncated versions of the WT SlMLO1 showed complete resistance to *O. neolycopersici* infection. Interestingly, the *Slmlo1* plants were free of any foreign T-DNA sequence and therefore were indistinguishable from natural *Slmlo1*-mutated plants (Nekrasov et al., 2017). An alternative approach for combatting powdery mildew disease is through the regulation of unicellular hyperresponsiveness (HR) in the penetration site of the fungi, and knocking out tomato Powdery Mildew Resistance 4 (SlPMR4) could make this possible (Santillan Martinez et al., 2020). SlPMR4 encodes an enzyme that catalyzes callose synthesis in response to environmental stress. Overexpression of PMR4 in *Arabidopsis* showed complete resistance to powdery mildew by blocking fungal penetration at the papilla sites (Huckelhoven, 2014). Surprisingly, *pmr4* mutants that do not produce callose at the papillae also resisted *O. neolycopersici* infection, which might have resulted from HR-like cell death at the infection site (Santillan Martinez et al., 2020).

##### Abiotic Stress Tolerance Engineering

CRISPR/Cas-based genome editing for abiotic stress tolerance in tomato breeding is promising for the creation of resilient cultivars for the sustainable production of tomato fruits. The most important abiotic stresses studied using CRISPR/Cas9 or Cas12a (Cpf1) tools in tomato have been drought (Wang et al., 2017; Li et al., 2019), salinity (Tran et al., 2020; Vu et al., 2020; Wang et al., 2020a,b) and temperature (Li et al., 2018b; Yin et al., 2018) (Figure 2 and Table 1). Two drought stress-responsive genes, mitogen-activated protein kinase 3 (SlMAPK3) (Wang et al., 2017) and non-expressor of pathogenesis-related gene 1 (SlNPR1) (Li et al., 2019), were knocked out by CRISPR/Cas9, but neither of them showed improvement in drought tolerance. The data indicate that SlMAPK3 and SlNPR1 may positively contribute to drought stress responses in tomato. Salinity-tolerant alleles were revealed from functional studies of genes relating to the perception of salt during plant growth. Another protein involved in K^+^/Na^+^ homeostasis in tomato is SlHAK20, a member of the high-affinity K^+^/K^+^ uptake/K^+^ (HAK/KUP/KT) transporter that was functionalized for salinity responses. The mutated *slhak20* allele contributed to the hypersensitivity to salinity (Wang et al., 2020b). Tomato Salt Overly Sensitive 1 (SlSOS1) is a Na^+^/H^+^ antiporter that helps to control Na^+^ levels in root epidermal cells. Blocking the activity of SlSOS1, therefore, reduced salt tolerance performance (Wang et al., 2020a). A very recent work conducted by our team revealed a strong salt-tolerant allele obtained by CRISPR/Cas9-based precise removal of a proline-rich domain of tomato hybrid proline-rich protein 1 (SlHyPRP1) (Tran et al., 2020). Another important environmental factor for the growth of tomato is temperature. Climate changes accompanying wider temperature changes may affect tomato cropping. Understanding the roles of genes involved in temperature responses is critical for engineering and breeding temperature-tolerant tomatoes. To this end, tomato C-repeat binding factor 1 (SlCBF1), a chilling-related gene, and Brassinazole Resistant 1 (SlBZR1), a heat-responsive factor, were knocked out by CRISPR/Cas9. The data showed that both genes were positively involved in temperature tolerance since the mutant alleles of *slcbf1* and *slbzr1* led to reduced chilling (Li et al., 2018b) and heat (Yin et al., 2018) stress tolerance, respectively. Further works are needed in order to reveal abiotic stress tolerance alleles for tomato breeding, especially those negatively affecting recessive alleles, or genome editing technologies to introduce dominant alleles.

#### CRISPR/Cas-Based Precise DNA Changes/Replacements

##### Base Substitutions

The uses of CRISPR/Cas complexes in tomato genome editing have not been limited to targeted mutagenesis but have been extended to precise changes of every base up to long DNA sequences. The direct evolution of nucleotide deaminases for fitting with CRISPR/Cas9-guides for base editing (BE) has been extensively conducted by Liu's group at Harvard University. For an extensive review, please refer to Ree and Liu's review (Rees and Liu, 2018). There are a limited number of published data applying base editors in tomato breeding. The *Petromyzon marinus* cytidine deaminase (PmCDA1) was fused to CRISPR/Cas9 [death Cas9 or nickase Cas9 (D10A)] for BE in tomato (Figure 1B). Efficient base substitutions in the Della gene led to a loss-of-function mutation that produced a *procera* phenotype with reduced serrated leaflets. Similar tools were also used for base substitutions in SlETR1, an ethylene receptor, to produce ethylene insensitive strains (Shimatani et al., 2017) (Table 1). In another paper, BE-based GE was used to produce herbicide tolerant tomato. Proline-197 in *Arabidopsis* ALS was shown to be the key a for conferring chlorsulfuron resistance when it was changed to another a.a. Thus, the corresponding proline-186 in tomato ALS1 was subjected to changes to other amino acids by nCas9-PmCDA1. Strains with substituted bases (C to T or C to G) exhibited strong resistance to the treatment of chlorsulfuron (Veillet et al., 2019).

##### Homologous Recombination (HR)-Based Knock-in

HKI in plants is an all-in-one precision technique for the replacement of SNPs or large DNA sequences (Figure 1A). However, due to the low efficiency of natural HR, the number of HKI applications in plants is limited and not currently available for practical use. With the advent of CRISPR/Cas complexes as molecular scissors for generating DSBs at specific genomic sites, HKI frequency has been improved dramatically (Cermak et al., 2015; Dahan-Meir et al., 2018; Merker et al., 2020; Vu et al., 2020). In tomato, HKI was engineered with CRISPR/Cas9 or Cas12a proteins and geminiviral replicons for generating anthocyanin overaccumulating events by inserting the CaMV 35S promoter upstream of SlANT1, an R2R3-MYB transcription factor. The HKI frequency was improved 10–30 times compared to that of the T-DNA cargos and was several orders of magnitude higher than that of spontaneous HKI (Cermak et al., 2015; Vu et al., 2020). A single amino acid substitution (N207D) in the polypeptide sequence of the *Arabidopsis* HIGH-AFFINITY K^+^ TRANSPORTER1 (AtHKT1) led to salt tolerance. Using the CRISPR/Cas12a-mediated HKI approach, we have successfully generated a salt-tolerant strain carrying a tomato ortholog of the AtHKT1 N207D, namely, SlHKT1;2 N217D, without using an allele-associated selection marker (Vu et al., 2020). CRISPR/Cas9-mediated HKI was also efficiently applied to generate herbicide-tolerant tomatoes by targeting SlALS1 for P186A modification (Danilo et al., 2019).

## Future Insights into Tomato Breeding

As discussed in the opening of this writing, the tomato domestication and selective breeding processes led to the reduction of genetic diversity in nowadays-cultivated tomatoes (Ranc et al., 2008; Lin et al., 2014). The cultivated tomato appears with high yield and compact architectures but tends to be more vulnerable to environmental attacks by physical as well as biological agents from the environment. Tomato production has become more difficult, especially in the face of global climate changes. Therefore, in the new scenario of tomato breeding, new margins of traits and techniques have to be fully considered. Alleles that determine abiotic and biotic stress-tolerant traits or fruit flavor have been widely lost in modern tomatoes but widely available in their wild relatives (Bai et al., 2018). Those alleles have been re-introduced into the cultivated tomatoes by conventional breeding without or with MAS. However, the traditional breeding approaches are time-consuming and laborious, especially for pyramiding polygenic traits or multiple monogenic traits of interest. The CRISPR/Cas-based *de novo* domestication and/or accelerated allele introgression could not only help to reduce the time and labor but also allow us to precisely control the genetic modification types (Fernie and Yan, 2019). Further, CRISPR/Cas-based pyramiding of polygenic traits or multiple monogenic traits could be feasible in a timely breeding program.

Another conventional approach in tomato breeding is random mutagenesis using the chemical as well as physical agents to disrupt the tomato genome and select for new alleles and acceptable mutant lines for further breeding purposes. Due to the random and extensive nature of the induced mutations, mutant lines show severely defective traits, and hence, cannot be suitable for crop production. Even if we can obtain a useful allele, its genetic background might have been dramatically changed from its parental origins. Thus, the approach is also time-consuming and laborious for the selection of usable alleles from a huge number of mutations. Again, CRISPR/Cas-based technologies appear to be revolutionized solutions for mining useful alleles for tomato breeding. The accumulated data showed high potential and efficient advances of multiplexed editing that can be used for discovering novel alleles based on the extensively released omics databases (Chen et al., 2019; Pramanik et al., 2020). With the multiplexed editing, engineering an intact metabolic pathway is also possible at high loci-specific precision (Li et al., 2018a,d). It is also wonderful to be able to obtain the homozygous edited alleles at the first generation of genome-edited events by haploid inducer-based genome editing (Kelliher et al., 2019). There are still hurdles in the selection and regeneration of edited events, especially those resulting from the allele-associated marker-free conditions in case of the low-efficiency HKI (Van Vu et al., 2019). Tackling the issue, some *in planta* transformation approaches mediated by *Agrobacterium* (Maher et al., 2020) or nanoparticles (Cunningham et al., 2018) could be applied for the genome editing process. Those approaches may help to significantly reduce tomato breeding time and labor that a small-scale enterprise can afford for contribution to the field.

### Accelerated Allele Introgression and *de novo* Domestication

**Figure 3:**
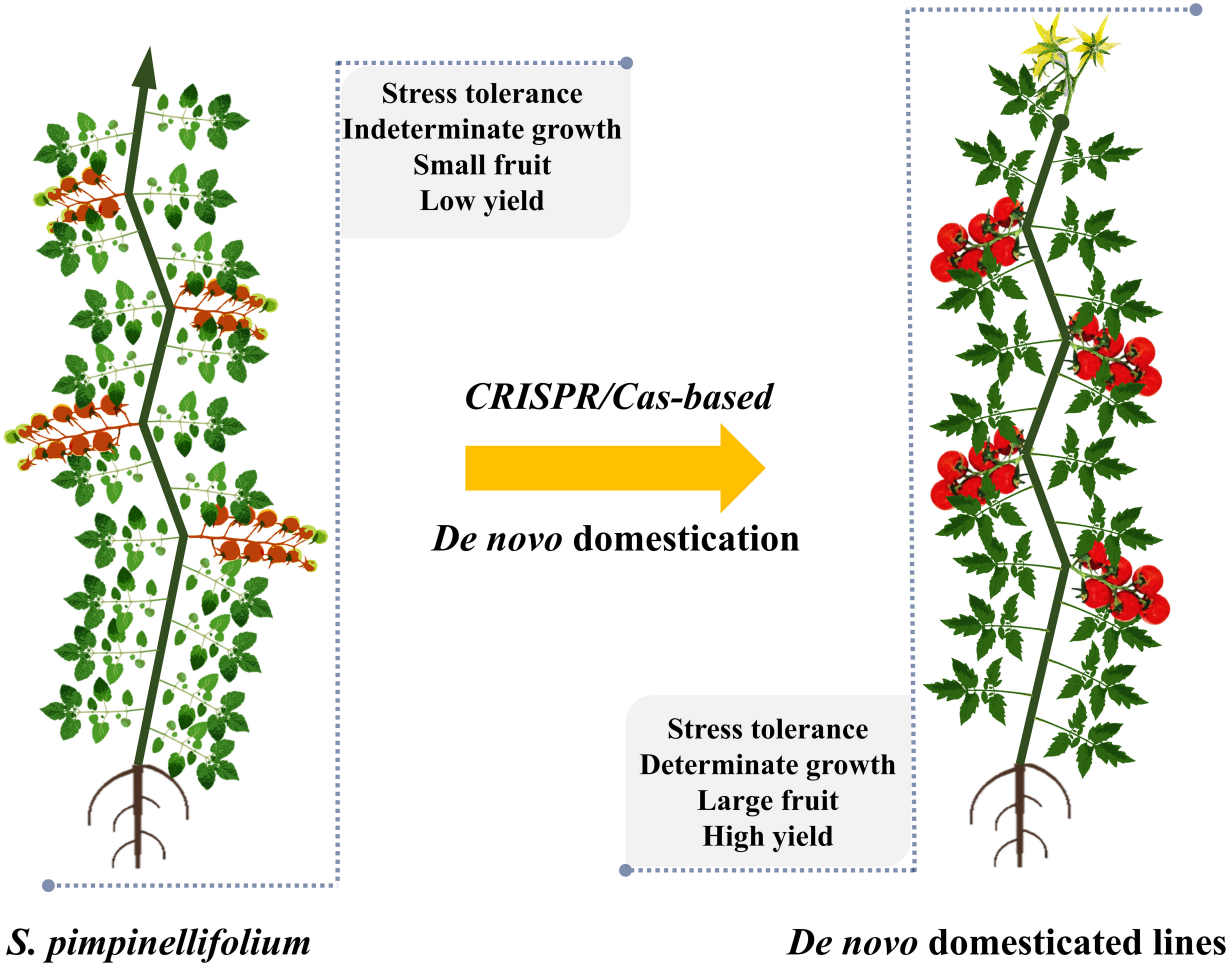
*De novo* domestication of *S. pimpinellifolium* by CRISPR/Cas technology. A representative *S. pimpinellifolium* plant and its *de novo* domesticated form are shown, and the CRISPR/Cas-mediated domesticated traits are indicated. The plants are not drawn to scale.

Food production with current technologies is predicted to not meet the demands of a dramatic increase in population by 2050 (Ray et al., 2013). Strategic breeding programs in the field have been initiated to reverse these catastrophic prospects, and integral solutions for sustainable agriculture will be key to overcoming food production barriers. The development of ideal/super crops should be a major goal (Zsogon et al., 2017). Engineering new crops by redomestication or *de novo* domestication from wild relatives or semidomesticated plants would also offer more options to cope with challenges in feeding people by 2050 (Fernie and Yan, 2019; Hickey et al., 2019). The most powerful applications of CRISPR/Cas technology in plant breeding may be the ability to accelerate the introgression of novel alleles into elite cultivars and the *de novo* domestication of wild plants for cultivation (Figure 3). The success of these processes is strongly dependent on the precision and efficacy of the CRISPR/Cas-based technologies. *De novo* domestication of wild tomato or orphan crops has been illustrated (Lemmon et al., 2018; Li et al., 2018c; Zsogon et al., 2018), thus paving revolutionary paths toward a new era of tomato breeding.

The lengthy domestication and conventional breeding processes have reduced some important qualitative and stress-tolerance traits (Liu et al., 2020; Wang et al., 2020a,b). Therefore, the *de novo* domestication of wild relatives or semicultivated plants by precisely introducing selected traits from their domesticated cultivars may yield potentially novel crops as alternative options for food supply. The ideas of redomestication and *de novo* domestication are strongly supported by CRISPR/Cas technology, which eases the manipulation of theoretically any genomic site of a genome of interest, including those of wild relatives and semicultivated plants. There are merely 15 plant species in use for food production, but thousands of semicultivated species are considered orphan crops and used to produce foods locally (Lemmon et al., 2018; Fernie and Yan, 2019). For tomato, the wild relative *S. pimpinellifolium* was used as a parental plant for *de novo* domestication of elite traits (Figure 3) (Li et al., 2018c; Zsogon et al., 2018). The most important traits selected for *de novo* domestication in the studies were growth habit (SELF PRUNING, SP and SP5G), fruit setting (OV, CLV3, FW2.2; MULT and WUS), and fruit quality (CycB and SlGGP1). The most desirable traits were obtained when knock-out mutations were precisely introduced in the selected genes in the genome of *S. pimpinellifolium* (Table 2) (Li et al., 2018c; Zsogon et al., 2018).

**Table 2:** *De novo* domestication of Solanaceae using multiplexed CRISPR/Cas tools.

**Species**	**Trait category**	**Target gene**	**Accession/Contig ID**	**Major mutant phenotype**	**Editing efficiency (%)**	**References**
*Solanum pimpinellifolium*	Growth habit	SlSP (Self-prunning)	Solyc06g074350	Determinate growth	30.0	Zsogon et al., 2018
	Fruit shape	SlOV (Ovate)	Solyc02g085500	Oval fruit	30.0	
	Fruit size	SlFAS (Fasciated/Yabby)	Solyc11g071810	-	0.0–66.7	
		SlFW2.2 (Fruit weight 2.2)	Solyc02g090730	No obvious phenotype	30.0–66.7	
		SlCLV3	Solyc11g071380	Higher fruit locule number and fruit weight	66.7	
	Fruit number	SlMULT (Multiflora)	Solyc02g077390	Higher number of fruits per truss	0.0–66.7	
	Fruit quality	SlCycB (Lycopene beta cyclase)	Solyc04g040190	Higher accumulation of lycopene	30.0	
	Growth habit	SlSP5G (Self-prunning 5G)	Solyc05g053850	Determinate growth	32.1–57.1	Li et al., 2018c
	Growth habit	SlSP	Solyc06g074350	Determinate growth		
	Fruit size	SlCLV3	Solyc11g071380	Very slighly higher locule number and fruit size		
	Fruit size	SlWUS (Wushel)	Solyc02g083950	Higher locule number and fruit size		
	Fruit quality	SlGGP1 (GDP-L-Galactose phosphorylase)	Solyc02g091510	Increased foliar ascorbic acid content	-	
*Physalis pruinosa*	Growth habit	PpAGO7	Ppr-t_75930 through Ppr-t_75944	Narrower leaves and petals	-	Lemmon et al., 2018
	Growth habit	PpSP	Ppr-t_24561	Severe determinate plant	-	
	Growth habit	pSP5G	Ppr-g_k141_ 668713	Determinate growth, higher fruit number	-	
	Growth habit	PpJ2 (Jointless-2)	Ppr-t_50452	Jointless unbranched inflorescences	-	
	Fruit size	PpCLV1	Ppr-t_75296	Higher locule number and fruit size	-	

Proposing a different strategy for *de novo* domestication using genome editing technology, Lemmon and coworkers introduced targeted mutations (orthologs of the tomato domesticated alleles) into the genome of an orphan crop (*Physalis pruinosa*), a sweet ground cherry of the *Solanaceae* originating from Central and South America. The determinate growth, higher fruit number, and higher locule number traits were precisely added into the plant (Table 2), and generated phenotypes similar to those of their tomato orthologs (Table 1) (Lemmon et al., 2018).

Taken together, the above studies have paved a novel path toward obtaining *de novo* domesticated/redomesticated plants at the fastest rates for the breeding of resilient tomato to cope with environmental changes by the wild genetic background while enhancing/sustaining productivity and fruit quality.

### More Precise Tomato Breeding at the “Speed of Light”

CRISPR/Cas-based targeted mutagenesis is highly flexible and efficient for targeting theoretically any desirable site. However, its precision is at the locus/gene level and is not controllable at every single base. Therefore, the approach can be readily applied for targeted knock-outs within coding sequences or random changes of non-coding sequences, such as *cis-*elements, for random promoter engineering (Rodriguez-Leal et al., 2017; Li et al., 2020).

Precision editing at every single base by base editors (Figure 1B) has been extensively conducted in animals and plants but is still limited to nucleotide transitions or C to G transversion. Most of the base substitutions shown in plants were [C/G to T/A] or [A/T to G/C] (Mishra et al., 2020), and transversion base editing has not been demonstrated in plants, thus limiting the applications of base editors for crop improvement. Nevertheless, BE has been used at limited scales in tomato (Table 1) (Shimatani et al., 2017; Veillet et al., 2019). Recently, novel approaches have been explored for substitutions of any base of interest, such as prime editing (PE) (Figure 1C), or precise editing at a medium DNA length using microhomology-mediated end joining (Tan et al., 2020; Van Vu et al., 2020). Although prime editor appeared to be efficient in animals (Anzalone et al., 2019) and well-adapted to monocot plants (Lin et al., 2020), its application in dicots remains limited and needs further improvement (Lu et al., 2020). While an allele that can be created by substituting just a few SNPs within a particular editing window is achievable by base editors or prime editors, the more base changes and the wider the DNA window that are required, the more complicated and challenging it is for base editors or prime editors to edit precisely (Rees and Liu, 2018; Hua et al., 2020; Lin et al., 2020; Mishra et al., 2020).

The HKI approach may be the last option for precise gene editing for crop plants due to its low frequency and complexity in design, but it can be used to precisely edit most, if not all, of the types of base/DNA changes of interest. HKI-mediated precision editing in tomato can range in size from a single base to thousands of base pairs (Cermak et al., 2015; Yu et al., 2017; Dahan-Meir et al., 2018; Danilo et al., 2019; Vu et al., 2020). HKI frequency has been continuously improved from the trace level in nature to a level that can realistically and affordably be used for crop precision breeding. The milestones in tomato HKI improvements came from the use of CRISPR/Cas for DSB formation (Yu et al., 2017; Danilo et al., 2019), the combination of CRISPR/SpCas9 with geminiviral replicons (Cermak et al., 2015; Dahan-Meir et al., 2018); and CRISPR/LbCas12a (LbCpf1) with multireplicons (Vu et al., 2020). HKI could be further improved by fine-engineering components of the CRISPR/Cas complexes, such as temperature-tolerant LbCas12a (Merker et al., 2020), to reach a true “speed-of-light” (Wolter et al., 2019) precision genome editing technology for tomato breeding.

## Concluding Remarks

The cultivated tomato was domesticated and selected to retain favorable traits for consumption and/or processing. However, the domestication process and subsequent breeding dramatically reduced the genetic diversity among the modern commonly used tomato cultivars (Lin et al., 2014; Liu et al., 2020; Wang et al., 2020a,b). Global and local climate change has put pressure on tomato growers to sustain production and, at the same time, to diversify their products, such as those with more favorable colors, flavors, or higher nutritional/health quality. Conventionally, to introgress an elite allele into a cultivated variety, breeders have to perform hybrid crossing with a donor source, usually a wild relative. The crossing helps to generate a hybrid genome with the allele of interest but also leads to the introduction of undesired genetic background or linkage drag from the donor parent. The most undesired traits can be removed by backcrossing several times to the parental elite line and selection for the interested allele in each generation of offspring. However, linkage drag makes this more challenging. Therefore, conventional breeding usually requires years to obtain a new tomato variety for cultivation, even with MAS approaches.

The emergence of CRISPR/Cas technology (Figure 1), one of the ultimate NPBTs, has spurred a revolution in crop breeding, including tomato breeding. CRISPR/Cas studies conducted on tomato as a model system have been extensively reported (Figure 2 and Table 1). Since then, allele introgression via targeted mutagenesis, as well as the more precise BE and HKI in tomato, has become easier, and the time required to produce a new variety has been vastly reduced, to months. CRISPR/Cas tools have been widely used for generating tomato strains with better growth habits, improved fruit quantity, and quality for higher productivity with better nutrition and health properties (Table 1). Moreover, research that focuses on the ability to withstand environmental stresses has also been extensively released (Table 1).

Recently, a new trend in using CRISPR/Cas in tomato breeding has been to accelerate the so-called *de novo* domestication of new tomato varieties by introducing elite traits evolved during domestication and selective breeding into its wild relative/ancestor *S. pimpinellifolium* (Figure 3 and Table 2). The resulting plants carried improved traits, such as better growth, larger fruits, and higher productivity and quality, but they still retain important wild traits, including stress tolerance, especially those traits determined by multiple genes/alleles (Li et al., 2018c; Zsogon et al., 2018). This approach would help to save years in breeding super tomato cultivars that are resilient to climate change. Another idea is to *de novo* domesticate tomato-like orphan crops through CRISPR/Cas-based introgression of orthologous genes/alleles of cultivated tomato for improved growth and higher yield (Lemmon et al., 2018). It would be interesting to apply this approach to many potential tomato-like orphan crops to increase the production capability of local growers.

CRISPR/Cas-based targeted mutagenesis itself is much more precise than random mutagenesis technologies using chemicals or radiation. However, a large portion of important traits in tomato is encoded by complex alleles that require precise base/sequence replacements. The recent advancement of CRISPR/Cas applications has created more precise editing tools, such as BE, PE, and HKI. These tools are continuously improving to become more efficient and precise for an era of faster tomato breeding.

## Author Contributions

TV and J-YK: conceptualization and supervision. TV: methodology and writing—original draft. TV, SD, MT, JH, and J-YK: writing—review and editing. JH and J-YK: funding acquisition. All authors contributed to the article and approved the submitted version.

## Conflict of Interest

The authors declare that the research was conducted in the absence of any commercial or financial relationships that could be construed as a potential conflict of interest.

## Abbreviations

BE: Base editing
cNHEJ: Canonical nonhomologous end joining
Cas: CRISPR-associated proteins
CRISPR: Clustered regularly interspaced short palindromic repeats
DSBs: Double-stranded breaks
HR: Homologous recombination
HKI: HR-based knock-in
NPBTs: New plant breeding techniques
NHEJ: Non-homologous end joining.
